# Inclusive engagement for health and development or ‘political theatre’: results from case studies examining mechanisms for country ownership in Global Fund processes in Malawi, Tanzania and Zimbabwe

**DOI:** 10.1186/s12992-019-0475-9

**Published:** 2019-05-07

**Authors:** Russell Armstrong, Arlette Campbell White, Patrick Chinyamuchiko, Steven Chizimbi, Sarah Hamm Rush, Nana K. Poku

**Affiliations:** 1 Health Economics and HIV/AIDS Research Division, University of KwaZulu-Natal, Durban, South Africa; 2 Bill & Melinda Gates Foundation, Seattle, Washington USA

**Keywords:** Global Fund to fight AIDS, Tuberculosis and malaria, Health financing, Country ownership, HIV/AIDS, Malawi, Tanzania, Zimbabwe

## Abstract

**Background:**

For many countries, including Malawi, Tanzania and Zimbabwe, 2017 was a transition year for support from the Global Fund to Fight AIDS, Tuberculosis and Malaria as one funding cycle closed and another would begin in 2018. Since its inception in 2001, the Global Fund has required that countries demonstrate ownership and transparency in the development of their funding requests through specific processes for inclusive, deliberative engagement led by Country Coordinating Mechanisms (CCMs). In reporting results from case study research, the article explores whether, in the context of the three countries, such requirements continue to be fit-for-purpose given difficult choices to be made for financing and sustaining their HIV programmes.

**Results:**

The findings show how complex, competing priorities for limited resources increasingly strain processes for inclusive deliberation, a core feature of the Global Fund model. Each country has chosen expansion of HIV treatment programmes as its main strategy for epidemic control relying almost exclusively on external funding sources for support. This step has, in effect, pre-committed HIV funding, whether available or not, well into the future. It has also largely pre-empted the results of inclusive dialogue on how to allocate Global Fund resources. As a result, such processes may be entering the realm of ‘political theatre,’ or processes for processes’ sake alone, rather than being important opportunities where critical decisions regarding priorities for national HIV programmes and how they are funded could or should be made.

**Conclusion:**

To address this, the Global Fund has begun an initiative to shore-up the capabilities of CCMs, with specialised technical and financial support, so that they can both grasp and influence the overall financing and sustainability of HIV programmes, rather than focussing on Global Fund programmes alone. What stronger CCMs could achieve, given the growing HIV-treatment-related commitments in these three countries, remains to be seen. Starting in 2020, the three countries will discover what resources the Global Fund will have for them for the 2021–2023 period. The resource needs for expanding HIV treatment programmes for this period are already foreseeable leaving few if any options for future deliberation should funding from the Global Fund and others not grow at a similar pace.

**Electronic supplementary material:**

The online version of this article (10.1186/s12992-019-0475-9) contains supplementary material, which is available to authorized users.

## Introduction

For most countries in the East and Southern African regions, 2017 marked another transition year in financial support from the Global Fund to Fight AIDS, Tuberculosis and Malaria (the Global Fund) as one three-year funding cycle closed, and preparations were completed for a new cycle to begin in 2018. This was certainly true for Malawi, Tanzania and Zimbabwe. These three countries have some of the highest burdens of HIV disease regionally and globally and, in terms of financial support for HIV programmes, receive among the largest Global Fund contributions in its world-wide grants portfolio for the disease.^1^ As it ends its sixteenth year of operation, the consensus remains that it is one of the most successful global health initiatives (GHIs) [1]. Across the African continent, Global Fund support has rolled back rates of preventable death due to HIV, TB or malaria; has strengthened important components of the health systems needed to deliver programmes and services; and has created and sustained partnerships between governments, civil society and the private sector, without which such gains in lives saved could not have accrued at such a rapid pace [2, 3].

What has distinguished the Global Fund from other GHIs are its core principles of partnership, country ownership, performance-based funding, and transparency, and its specific requirements for ensuring alignment with these principles by the countries it supports [4]. In many respects it has defined itself as a ‘hands-off’ financing entity allowing recipient countries a significant amount of latitude to determine funding priorities and implementation modalities. Country-owned and country-led has been a main component of the Global Fund’s approach since its inception in 2001 [4, 5]. In starting out this way, the Global Fund was responding to a particular historical moment in the global discourse on aid effectiveness as well as mirroring the characteristics of the global AIDS response at the time, particularly the prominent role demanded by non-state actors as well as by people living with HIV (PLHIV) themselves [5, 6]. For close to two decades now these central features have remained and there are specific guidelines and policies for how they should be realised by recipient countries. These are largely in the form of prescribed structures and processes for inclusion and deliberative engagement across the different entities and sectors that are involved in national responses to one or more of the three diseases—HIV/AIDS, tuberculosis or malaria. Indeed, evidence that these things occur is a requirement of eligibility for countries to request for Global Fund support in all but exceptional cases [7].

Over the time that such requirements have been in place, however, the situation of these global epidemics, particularly for HIV, has evolved dramatically [3]. Such is the case currently for many countries within the East and Southern African regions, including for Malawi, Tanzania and Zimbabwe. ‘Hard choices’ are becoming the norm as these states must grapple with a growing tension between HIV epidemics that continue to expand, fragile health systems, and flatlining financial resources which mostly come from external sources [1]. The dominant strategy for managing HIV epidemics has become expanding anti-retroviral treatment (ART) programmes with a commitment to enrol at least 90% of individuals diagnosed with HIV disease on ART by 2020 [8]. While this push for high ART coverage has contributed to decreases in the annual number of new HIV infections and AIDS-related deaths globally, including in the three countries that are the subject of this article, enrolling individuals on ART is a lifelong commitment for which competent health systems need to be in place and sufficient financial resources be available well into the future. There is mounting pressure, then, between this drive to support as many individuals as possible on ART and the increasingly uncertain prospect of sufficient resources being available to finance this approach for as long as the number of adults and children needing treatment continues to increase [9].

Such ‘hard choices’ have naturally become central topics of concern within country-level structures and processes for planning and prioritising Global Fund investments. It is reasonable to ask, therefore, whether they continue to be fit-for-purpose given the magnitude of these challenges. Country experiences with the recently completed funding renewal cycle present opportunities to examine this question. Although there is an increasing amount of scholarly attention to this topic, very little has focussed on the East and Southern African regions [10–12]. This article seeks to address this gap by reporting on exploratory research on Global Fund processes conducted in Malawi, Tanzania and Zimbabwe as they prepared and submitted new Funding Requests during 2017. The findings demonstrate how such ‘hard choices’ are placing increasing amounts of strain on these processes, particularly the components for multi-sectoral inclusion and deliberative engagement, core requirements for demonstrating country ownership. Burgeoning resource needs for ART programmes and how to address them may be pushing such procedures towards the realm of ‘political theatre,’ or processes for processes’ sake alone, rather than being important platforms or mechanisms, as they were originally intended, where critical decisions regarding programmatic and financial priorities for national HIV programmes could or should be made [6, 12].

## Research aims and methods

This article draws on findings from a set of case studies that examined how Malawi, Tanzania and Zimbabwe planned for, received and structured their Global Fund investments in HIV programmes. The studies were commission by the Bill & Melinda Gates Foundation as broadly descriptive analyses of Global Fund structures and processes in each country where the foundation was seeking to deepen its engagement with all stakeholders in order to improve the effectiveness and impact of national HIV responses. The research was conducted between 2017 and 2018. The results from each case study were used to highlight opportunities for the foundation and for other technical partners to support optimisation of Global Fund processes with a view to accelerating progress towards national, regional and global level targets. These targets primarily aim to prevent, to the greatest extent possible, new HIV infections and to reduce AIDS-related deaths. As potential opportunities for optimisation, the case studies paid specific attention to: participatory and inclusive mechanisms for governance, oversight and accountability; how prioritisation occurs in terms of decisions about what Global Fund investments should support; the positioning of these investments within the health sectors; and the extent of linkages or complementarities with other mechanisms for the governance and management of development assistance for health in these three countries.

Following a typical case study approach, data collection involved desk review, key informant interviews, and participant observation in meetings and workshops. The desk review component encompassed peer-reviewed literature, programme and meeting reports, technical guidance documents, Global Fund Funding Request submissions, other Global Fund documentation, and a variety of other relevant sources. Key informant interviews, using a semi-structured topic guide, were conducted with members of Country Coordinating Mechanisms (CCMs); key officials in government ministries; representatives from civil society organisations, including those representing important constituencies such as people living with HIV (PLHIV) and key populations (men-having-with-men or sex workers, for example); health development partners, particularly the United States President’s Emergency Plan for AIDS Relief (PEPFAR); representatives from United Nations agencies, particularly, the Joint United Nations Programme on HIV/AIDS (UNAIDS) and the World Health Organisation (WHO); and, to a more limited extent, representatives from the Global Fund itself, particularly members of Country Teams. In total, 81 individuals participated in interviews across the three coutries.

Researchers attended and observed numerous meetings and workshops, particularly during Country Dialogues and the development of Funding Request submissions. In two countries, Malawi and Tanzania, researchers also served as technical experts supporting Funding Request development. Data analysis involved thematic analysis of documents and interview notes, basic statistical analysis of financial data, and triangulation. Country-specific case study reports were shared with CCMs and other relevant stakeholders for validation prior to finalisation and release.

## Results

### Country ownership, deliberative engagement and what they entail

As Global Fund structures and processes that embed country ownership have evolved over time, one constant has been the requirement that countries establish and maintain CCMs [4, 13]. These are multi-sectoral bodies comprised of representatives from ‘constituencies’ or sectors. Government ministries, academic institutions, international and national non-governmental entities, private sector entities, and organisations involving people living with or affected by HIV, TB or malaria are among the ‘interested’ parties that participate in such structures. They are meant to be the vehicle for stakeholder collaboration and participatory decision-making regarding Global Fund investments. As the Global Fund itself has stated, “CCMs are central to the Global Fund’s commitment to local ownership and are a ground-breaking, innovative mechanism towards stakeholder collaboration and participatory decision-making [14].” They are also meant to take into account and to influence the broader landscape of health programming and financing in their countries to ensure that Global Fund investments leverage and achieve synergies with other domestic and external support to the health sector [4, 13].

In Malawi, Tanzania and Zimbabwe, CCMs have been in place for nearly two decades and although they may have a functional link with a statutory body, such as the Office of the Prime Minister in Tanzania, they remain largely ad-hoc entities structured and managed according to the criteria and guidelines issued by the Global Fund [13]. These are creatures of policy rather than law, in many respects, and they are tasked with monitoring hundreds-of-millions-of-dollars-worth of Global Fund investments in critical health programmes. Membership ranges from seventeen representatives in Malawi to twenty-two in both Tanzania and Zimbabwe. There are an equal number of alternate members in each country. During 2017, the three CCMs were actively engaged in participatory and deliberative processes for the development of their most recent round of Funding Requests to the Global Fund. However, questions arose regarding the relevance and effectiveness of much of these efforts given the existence of important prior constraints, in the form of large and growing ART programmes, which substantially limited the field of deliberation and the outcomes that resulted in terms of the final contents of the Funding Requests themselves. In order to understand how this situation arose, and the extent to which it poses a growing challenge for this important feature of Global Fund’s approach, it is important to firstly outline the country settings in which these processes unfolded, including the magnitude of their HIV epidemics and the status of current efforts to address them.

### Country contexts for Global Fund programmes

How GHIs such as the Global Fund function to deliver health benefits is bound up with important features of the country contexts in which they operate. In the cases of Malawi, Tanzania and Zimbabwe, these are characterised by a number of social, economic and health-related challenges that surround Global Fund programmes with numerous competing priorities. Table 1 provides some comparative data on these issues:

**Table 1:** Relevant Features of Country Contexts (2017)

Component	Malawi	Tanzania	Zimbabwe
Population [31]	18.6 million	57.3 million	16.5 million
Young age (0–14 years) dependency ratio (per 100 population 15–64 years) [31]	82.9	86.4	73.6
HDI rank [31]	171	154	156
Poverty (% with <US$1.90 PPP) [31]	71.4%	49.1%	21.4%^a^
Leading causes of death—all ages (in rank order) [32]	HIV; lower respiratory infections; malaria; diarrheal diseases	HIV; malaria; lower respiratory infections; diarrheal diseases	HIV; lower respiratory infections; diarrheal diseases; TB.
Income status [33]	Low	Low	Low

^a^There is some debate about the accuracy of this data. A clearer picture emerges when considering that of the 79.3% of the adult population that was employed in 2017 (including self-employment and unpaid domestic work), 74.8% earned US$3.10 PPP or less [31]

All three countries have very young populations with very high dependency ratios. For Tanzania, this reaches as high as 86 dependent children for every 100 working-age adults across the population. It is very important, then, how each country manages its HIV epidemic, particularly for the prevention of new HIV infections amongst young people, as these measures will have a future bearing on population-wide sexual and reproductive health (SRH). All three countries fall within the least-developed-country grouping, which is the bottom third of the 180-country Human Development Index. They have significant rates of severe poverty which are most acute for Malawi and Zimbabwe. For the East and Southern African regions, poverty is a main structural driver of HIV epidemics, particularly for adolescent girls and young women [15]. It is also a key factor affecting adherence to ART [16]. HIV remains the leading cause of disability and death in all three countries. Finally, these countries’ inclusion within the World Bank’s low-income grouping positions them to both need and to be eligible to receive substantial amounts of external assistance for HIV and other health priorities with, in the case of the Global Fund, very low requirements for raising counterpart domestic contributions through the national budget [17].

### Status of HIV epidemics and progress of national HIV programmes

One of the indicators of need for this external support for HIV programmes is the magnitude of the burden of disease that each country experiences as well as the progress each one makes in terms of improving this situation. To illustrate this, Table 2 shows comparative data on selected HIV-related characteristics.

**Table 2:** Selected Indicators for HIV Responses in Malawi, Tanzania and Zimbabwe (2017) [18]

Indicator	Malawi	Tanzania	Zimbabwe
Adult (15–64 years) HIV prevalence	10.6%	5%	14.6%
Number of PLHIV (all ages)	1,444,163	1,446,355	1,325,823
New HIV infections in 2017 (all ages)	38,650	65,285	40,973
Percent change in new HIV infections since 2010	− 40%	− 22%	−44%
Proportion of PLHIV (all ages) with viral suppression	61.4%	48%	60%

Although HIV prevalence is lower in Tanzania than in the other countries, the size of its population (57 million in 2017) means that in absolute numbers it has one of the larger HIV epidemics in the region. There are sizeable populations of PLHIV in all countries which are continuing to grow. Although the annual number of new infections has continually declined since 2010 (along with the numbers of AIDS-related deaths which are not shown), largely due to the significant increase in coverage of ART over the same period, their number continues to be significant, reaching as high as 65,000 in Tanzania in 2017 [3, 18]. While all countries, in line with WHO guidance, have adopted the ‘test and start’ approach, which means that PLHIV are eligible to start ART upon diagnosis, the proportion of PLHIV enrolled on ART to the extent of reaching viral suppression (and minimising the risk of onward HIV transmission) reaches only as high as 61% in Malawi among the three countries [18, 19]. Sustained efforts are still required, then, in order to increase this proportion, particularly when the need for ART is still expanding at the rate of tens of thousands of individuals newly infected with HIV each year.

### Sources of funds for HIV programmes

**Fig. 1:**
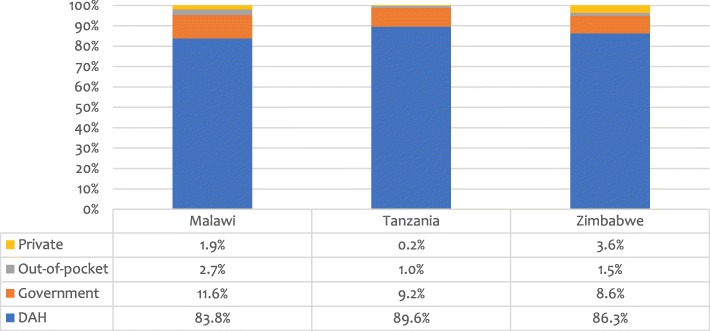
Proportional sources of funding for HIV programme expenditure [20]. DAH = Development assistance for health

How are these efforts paid for and sustained? In the three countries examined here, HIV programmes are largely externally funded as they are in most countries across the East and Southern African regions [20]. Figure 1 shows proportional sources of funding for national HIV programme expenditures.

According to these data, government support for HIV programmes was only 12% in Malawi and approximately 9% in Tanzania and Zimbabwe in 2017. External financiers, primarily the Global Fund and PEPFAR, made up the balance, contributing as much as 89.6% of total funding for HIV in Tanzania [20].

**Fig. 2:**
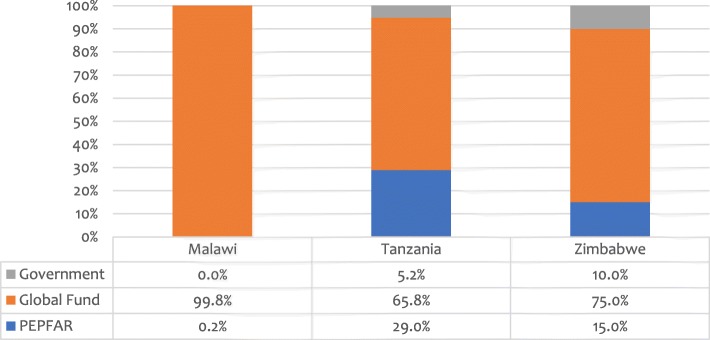
Funding of ARVs by source [22, 24, 25]. Note: For financial data, see Additional file 1: Table S1

With regard to the specific component of support for anti-retroviral medicines (ARVs), as of 2016, Global Fund remained the primary funder in each country as Fig. 2 illustrates.

Domestic financial support for ARVs is minimal at best, at 0% in Malawi, 5% in Tanzania and 10% in Zimbabwe. In 2017, the number of PLHIV receiving ART was 740,000 in Malawi, and 965,000 and 1,120,000 in Tanzania and Zimbabwe, respectively [18]. The fact that financing for ARVs for these individuals was almost exclusively dependent on external sources raised questions about the stability and sustainability of ART programmes for the hundreds of thousands of adults and children that now depend on them in these countries [1, 9, 20].

### Contributions of Global Fund processes: leading or following?

As the planning for new Global Fund funding cycles began in 2017, these were the ‘starting positions’ for each country: complex, aid-dependent environments with substantial socio-economic and health related challenges; expanding HIV epidemics; a strategic approach to HIV epidemic management that prioritises scaling-up of ART over other options such as primary prevention programming; and an almost exclusive reliance on external sources, primarily the Global Fund and PEFPAR, to finance these efforts. In keeping with the country ownership principle, the Global Fund stipulates that the development of Funding Requests must also be inclusive and deliberative through the requirement that CCMs convene Country Dialogues. These are defined as “… an ongoing process that occurs at the country level amongst all stakeholders...to develop and revise health strategies to fight the three diseases and strengthen health and community systems [7].” Evidence that Country Dialogues have occurred is a required component of Funding Request submissions without which they will not be accepted by the Global Fund [7].

As the case studies showed, these deliberative processes took place in each country although to varying degrees of scale and scope. Zimbabwe, for example, had the most extensive process with a series of consultations varying in both thematic focus and geographic location lasting over a period of months and involving hundreds of participants. The process was less elaborate in Tanzania and Malawi. However, in all cases, they were not continuations of existing processes examining “health strategies” or “strengthening community systems [7].” They were, rather, specially convened for the purpose of supporting the development of the Funding Request. And while many key informants who participated in them were of the view that these deliberations were important, a number questioned the outcomes as the lengthy lists of ‘priorities’ they generated for inclusion in the Funding Request ultimately exceeded the amounts that the Global Fund had made available.^2^ To some they appeared to be ‘wish-lists’ rather than carefully considered, essential items of critical value for their national HIV responses. In no country were detailed financial data shared at the start of these deliberations regarding needed investments to support ART programmes and the limited opportunities available within the Global Fund allocation to support other priorities once these needs were addressed.

Following on from the County Dialogue stage, CCMs then engaged groups of expert consultants to lead writing teams to prepare the Funding Request submissions. Large writing teams, involving more than 100 participants in Tanzania and Zimbabwe, for example, worked through an iterative process covering weeks or months to winnow the expansive lists of ‘priorities’ into final sets of items that fell within the available funding allocations, or that could be included as part of the Prioritised Above Allocation Request (PAARs) components.^3^ How such choices were made in each country was not always transparent as several key informants voiced concerns that Ministries of Health or the technical consultants themselves appeared to be the primary decision-makers, although the CCMs also played a role in reviewing and approving iterations of both the Funding Requests and the PAARs.

**Fig. 3:**
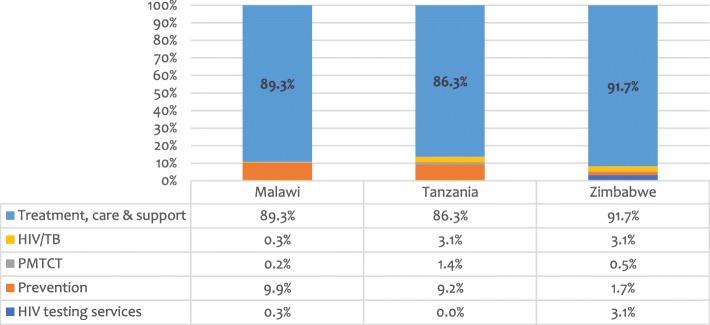
HIV funding allocations by programme area. PMTCT = prevention of mother-to-child transmission of HIV. Note: For financial data, see Additional file 1: Table S2

When the final submitted versions of the Funding Requests were examined, more questions arose regarding what such extensively participatory and deliberative processes achieved. As Fig. 3 illustrates, across each main funding allocation for HIV (excluding other components such as for TB or health systems strengthening), almost all of the available funding was budgeted for ART programmes, a result that may appear to have been a foredrawn conclusion even before the Country Dialogue processes began.

Treatment programme budgets took up 92% of the main HIV allocation for Zimbabwe; for Malawi and Tanzania, the proportions were 89 and 86%, respectively. The proportion budgeted for prevention was, correspondingly, minimal at only 1.7% in Zimbabwe, but higher in Malawi and Tanzania, at 10 and 9%, respectively. Additional amounts were available for specific prevention priorities in each country under “catalytic” or “matching fund” arrangements which will partially explain the low proportions in the main funding allocations [21]. These included, for each country, between US$7–8 million in additional support for programmes addressing HIV prevention and other needs for adolescent girls and young women, and, for Zimbabwe, an additional US$10 million for prevention programmes addressing key affected populations, including for sex workers and men-having-sex-with-men. This changed the picture to some extent and raised the proportional investment in HIV prevention by an additional 5% for Zimbabwe, and 2–3% for Malawi and Tanzania.

**Fig. 4:**
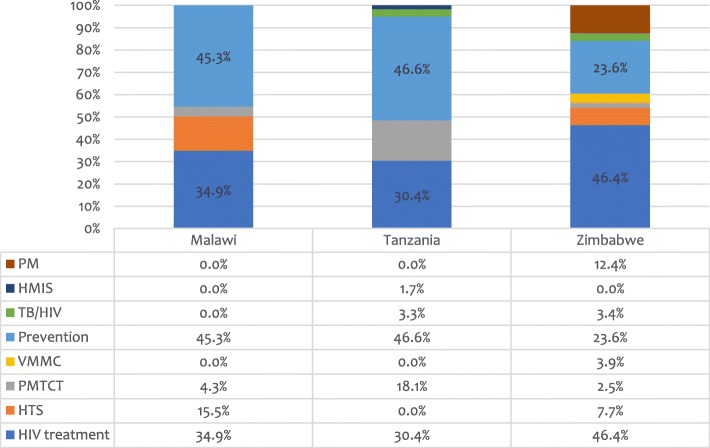
Proportional allocation of PAAR amounts by programme category. PM = programme management; HMIS = health management information systems; VMMC = voluntary medical male circumcision; PMTCT = prevention of mother-to-child transmission of HIV; HTS=HIV testing services. Note: For financial data, see Additional file 1: Table S3

When the contents of the PAARs for each country were examined, the analysis showed how, in addition to more treatment needs, there was also inclusion of allocations for interventions for HIV prevention. Figure 4 below gives the proportional allocation of the total PAAR amounts by programme category.

For Zimbabwe, from a total PAAR of US$144 million for HIV interventions, 46% or US$66 million was allocated for additional HIV treatment needs. The proportion was much smaller in Malawi and Tanzania but still constituted approximately one third (35 and 30%) of the total request. Also, in these two countries, allocations for prevention were substantial at almost half of the total request (45 and 47%), primarily for programmes for key populations and other vulnerable groups. As noted above, the lower allocation for prevention in the Zimbabwe PAAR was likely due to the availability of matching funds for this purpose. While, overall, the inclusion of prevention needs within the PAARs reflected how this critical programme component was still prioritised in the Funding Request process, there are some risks associated with this move. PAARs are to be financed through different means, primarily through reallocation of unused or surplus funds over the course of the three-year grant implementation period [7]. There is no guarantee, then, at the outset of a funding period that such opportunities will arise, at least to the extent that a substantial amount of the PAAR requests will be addressed.

There were some additional aspects to this situation to consider. As already noted, PEPFAR contributions are also substantial, reaching as high as 67% of all external resources for HIV programmes in Tanzania in 2016, for example [22]. Country planning for PEPFAR support is separate from Global Fund processes although the two are necessarily linked. PEPFAR processes take place annually and result in one-year Country Operational Plans rather than the Global Fund’s three-year funding agreements [23]. The amounts involved for the 2017/2018 year were large, ranging from US$132 million for Zimbabwe to US$433 million for Malawi [22, 24, 25]. The focus for these investments has become “total epidemic control” with the main priority being to, “optimize identification of PLHIV, linkages to services, uptake of ART, retention, and adherence [26].” While PEPFAR resources also support certain prevention interventions, such as voluntary medical male circumcision, prevention of mother-to-child transmission of HIV, and, to a more limited extent, sexual behaviour change, these generally have a lower priority and receive a lesser share of available resources [22, 24, 25]. Even with the addition of PEPFAR investments, which help to fill the crucial gap between what Global Fund provides and the total resource needs for HIV programmes in the absence of substantial domestic contributions, much of available funding was still prioritised towards increasing ART coverage.

Finally, even after taking into account these substantial commitments of external resources, funding gaps remained. There are two examples of what these gaps entailed. A resource needs analysis conducted in Tanzania in 2015 showed that, in order to reach the 2020 Fast-Track targets of 90% of diagnosed PLHIV being enrolled in ART, the annual funding gap would reach approximately US$180 million should substantial increases in either Global Fund or PEPFAR support not occur and the level of domestic contribution not change [27]. For Zimbabwe, this figure was estimated at more than US$200 million by 2020 [28]. Each country has set out plans for how to address these gaps through, in part, increased domestic resource mobilisation. These plans need to deliver results quickly, however, and reach US$100–200 million per year by 2020. Both countries remain optimistic, but the challenge ahead is significant given the large distance between these goals and the current situation of relatively small domestic financial allocations.

## Discussion--inclusive engagement or ‘political theatre’?

Global Fund programmes in Malawi, Tanzania and Zimbabwe, unfold within larger country contexts characterised by high levels of foreign aid dependence, particulalry for the health sector, and multiple and competing health and development priorities for their primarliy young and largely impoverished populations. In spite of these complexities, though, when coupled with support from PEPFAR, the Global Fund’s investments in national HIV programmes have contributed to substantial reductions in new HIV infections and AIDS-related deaths. However, HIV epidemics are continuing to expand in each country despite such progress. There is a primary focus on achieving high coverage of ART as the main approach to epidemic management, in line with the global emphasis on reaching 90% of diagnosed PLHIV with this life-saving intervention by 2020. Few if any domestic resources are used to support the direct costs of such plans, particularly for ARVs. Constraints on the amount of external funds available have created an increasing tension between the drive to continuously expand ART coverage and the reality that, at least by 2020, projected costs will exceed the supply of all resources, whether domestic or foreign, to a significant degree. The potential for investment in other strategic options for managing HIV epidemics, including primarly prevention programmes, is significantly limited in this context in the absence of new funding sources. There is more and more attention to the risks of this approach. Global experts have been repeating with increasing volume that 2030 targets to end HIV as a public health threat will not be achieved through high ART coverage alone, even if it were to be attainable and sustainable in all settings [1, 3].

What comes next, once the current Global Fund grant cycle ends, and in the absense of any sign at this point that additional resources will be available to address predicted funding gaps, none of the three countries has clearly set out. Nor, given the findings from the case studies, were there any indications that this topic was broached, either as part of Country Dialogue or as part of the Funding Request development process. What apprears to have preoccupied the participants in these deliberations is how to distribute the Global Fund allocation across different ‘priorities’ with the end result being that ART needs dominated in the main allocations with some additional items related to prevention and other non-HIV-treatment needs being included in the PAARs or as part of PEPFAR support. There was little evidence of any debate about the more fundamental questions of the stability and sustainability of national HIV responses that rely almost exclusively on increasingly static amounts of external resources, something over which CCMs and the countries themselves appear to have little substantive influence. They can react to and plan for what funds they are allocated. However, since the Global Fund changed its appraoch in 2014, and taking into account that PEPFAR allocations have always been centrally determined in the US, there are few if any opportunities to push for more [7, 28]. And, one may argue, it is difficult to see what the potential strength would be in such a negotiating position when, as with Malawi and Tanzania, for example, after more than a decade-and-a-half of Global Fund support (amounting to US$677 million for HIV in Malawi, and US$1.2 billion in Tanzania) domestic contributions to HIV programmes remain minimal at best.

What did inclusive and deliberative engagement through Global Fund processes contribute to this situation? In the view of the Global Fund, and amongst a number of the stakeholders interviewed as part of the case studies, while CCMs are meant to provide a platform for tabling and debating such issues of growing magnitidue and complexity, they are not fulfillling this function. Their work, as this analysis has shown, has increasingly been about allocating support for ART programmes, an outcome that is more or less foreseen given how funding requirements for current or future treatment needs can be calculated fairly discretely without the requirement of inclusive dialogue. To recall at this point that, in each of the three countries, Country Dialogues and Funding Request development processes unfolded over several weeks and months involving hundreds of participants is to highlight a growing disconnection between broadly deliberative processes and the substance of what the processes are meant to address. While suggesting that such efforts are entering the realm of ‘political theatre’ may be too strong a statement for some, it does serve to draw attention to this growing grap.

## Conclusion--who will decide?

Global Fund processes, as they have been designed, are meant to serve important ends in the form of national conversations and multi-sectoral engagement on critical questions and issues arising within countries for the health of their populations. These have to do with how to design and implement interventions as HIV epidemics continue to evolve and become more complex, and how to finance these activities to the scale and scope required for countries to fulfil national and global HIV-related commitments for 2020, 2030 and beyond. However, what these processes can achieve, given the increasingly narrow scope for decision-making in the context of large and continually expanding ART programmes, raises important considerations about their relevance and effectiveness, at least in their current form as they have been outlined by the Global Fund and as countries have interpreted and applied these requirements.

What the analysis has also raised is the reality that financial planning for national HIV responses, and the decisions that are made about where the increasingly finite amount of resources are directed, has become complex and highly charged. While, for example, all three countries duly met requirements for inclusive processes for Country Dialogue and Funding Request development, how Funding Requests were shaped, particularly how the allocations were distributed across programme areas, appeared in the end to be fixed in advance or at least much less open to input from this engagement. Ministries of Health receive the biggest share of Global Fund resources because they are responsible for ART programmes; technical experts are needed to forecast and cost current and future ART needs; and funders like the Global Fund and PEPFAR favour ART scale-up in their funding strategies. It is difficult to challenge a desire to offer as many PLHIV as possible life-saving ART; however, unless the fundamental formula of how this approach is financed begins to change, difficult choices clearly loom during and beyond the 2018–2020 period should the growing cost of ART programmes subsume most if not all of the available domestic or external financing while still leaving a significant funding gap [1, 9, 20]. Certainly, for Malawi, Tanzania and Zimbabwe, these options for change may be few given the competing demands of other health and development needs on national budgets.

Are CCMs equipped to navigate these issues using Global Fund processes for inclusion and deliberative engagement? And if not through these structures and processes, where will such issues be addressed? To the extent that such decisions should not just involve government actors, the case studies showed that despite their limitations, the CCMs in each country were the more functional multi-sectoral entities that could potentially address such questions of national concern. The Global Fund itself concurs, although not without acknowledging the need for some improvements. While its own reviews carried out in 2016 affirmed the importance of country ownership and the CCM role, they also identified some weaknesses, particularly a lack of technical capacity amongst members on many CCMs to fulfil the demands of their role, and a lack of strategic engagement or integration of these entities with other important national mechanisms for policy-making, planning and decision-making for health and development [14, 29].

To address these challenges, the Global Fund has launched a new CCM strengthening initiative beginning with a group of 18 pilot countries, of which Tanzania is one [30]. Two priorities for the initiative are: improving the capacities of CCMs to both grasp and influence the ‘bigger picture’ for financing and sustaining national HIV responses; and offering technical support for CCMs to work to embed themselves more permanently within the national architecture for health and development planning and oversight. Something that may be absent from the plan is critically reflecting on the extensive nature of the ownerhsip and inclusion requirements and a consideration of scaling them back, or making them less grandiose, given the country level realities this discussion has explored. What influence CCMs can have, even with stronger capacity, over the relatively entrenched direction of most national HIV responses, particularly in Malawi, Tanzania or Zimbabwe, remains to be seen. Time will tell over the near future as the next Global Fund replenishment drive takes place during 2019 and, subsequently, countries discover what resources it will commit to them through the 2021–2023 period and afterwards. Should such commitments continue to not increase, and bold plans to achieve efficiencies and raise additional domestic resources fail to bear sufficient fruit, even ‘harder choices’ may arise for CCMs that will further strain the inclusive and deliberative processes that they are meant to lead.

## Limitations

Many different individuals and entities were involved in Global Fund processes in each country. Not all were available for interview during the time that the case studies took place. As a result, some important viewpoints may not have been fully represented in the analysis. CCMs are not the only arena where priorities for national HIV responses, including how they are funded, are debated and decided upon (government Cabinets, for example, may play a more central role) so the analysis may risk overstating their part to address the difficult choices the three countries may be facing. Finally, the study has focussed on only selected elements of the more elaborate nature of the partnership between the Global Fund and the countries it supports. How the issues identified in this analysis link to and may be mediated by these other features would be the subject of a much broader inquiry.

## Additional file

Additional file 1: File contains three supplementary tables showing detailed financial data from which percentage calculations in Figs. 2, 3 and 4 in the main manuscript were derived. (DOCX 55 kb)

## Footnotes

1 For the 2017–2019 period, the available amounts were US$408 million (Tanzania), US$406 million (Zimbabwe), and US$318 million (Malawi). These countries also receive funds for TB and malaria programmes, the amounts for which are not included in this analysis but are much lower. See: https://www.theglobalfund.org/en/funding-model/funding-process-steps/allocations/. Accessed: 8 Jan 2018.

2 Since 2014, the Global Fund has calculated on its own the total funds it is willing to offer a recipient country and informs the CCM of this amount in writing as the Fund Request development cycle begins. Countries are recommended to address their core HIV fundings needs within this allocated amount [7].

3 Since 2014, the Global Fund has allowed its recipient countries to submit funding requests that exceed what it has nominally committed to provide through its main funding allocations. In the 2017 funding cycle, these were called Prioritised Above Allocation Requests. The reason for this is to allow countries to demonstrate that effective management of their epidemics requires additional investments. The Global Fund approves the technical merits of these additional requests and commits that, should additional resources become available over the three-year funding cycle, some of these additional items may be funded [7, 34].
