# Supplementary material for: Inclusive engagement for health and development or ‘political theatre’: results from case studies examining mechanisms for country ownership in Global Fund processes in Malawi, Tanzania and Zimbabwe
Source: Global Health. 2019 May 7;15:34. doi: 10.1186/s12992-019-0475-9 (PMC6505082; doi:10.1186/s12992-019-0475-9)
Supplement: Supplementary file 1 — File contains three supplementary tables showing detailed financial data from which percentage calculations in Figs. 2, 3 and 4 in the main manuscript were derived. (DOCX 55 kb) [file 12992_2019_475_MOESM1_ESM.docx]

**Additional file 1: Table S1: Analysis of Funding Sources for ARVs (2016)**

| **Source of Funds** | **Malawi (US$)** | **%** | **Tanzania (US$)** | **%** | **Zimbabwe (US$)** | **%** |
| --- | --- | --- | --- | --- | --- | --- |
| PEPFAR | 283 061 | 0% | 51 713 213 | 29% | 19 436 010 | 15% |
| Global Fund | 141 530 527 | 100% | 117 431 937 | 66% | 97 180 051 | 75% |
| Government | - | 0% | 9 233 581 | 5% | 12 957 340 | 10% |
| Other | - | 0% | - | 0% | - | 0% |
| **Total** | **141 813 588** | **100%** | **178 378 731** | **100%** | **129 573 401** | **100%** |

**Table S2: Analysis of Global Fund Funding Request Allocations for HIV**

**Legend:** FSW=female sex workers; HMIS=health management information systems; M&E=monitoring and evaluation; MSM=men-having-sex-with-men; PMTCT=prevention of mother-to-child transmission of HIV; PWID=people who inject drugs; RSSH=resilient and sustainable systems for health; VMMC=voluntary medical male circumcision.

| **Malawi** |  |  | **HIV only** |  |  |
| --- | --- | --- | --- | --- | --- |
|  |  |  |  |  |  |
|  |  |  |  |  |  |
| **Module** | **US$** | **%** | **Module** | **US$** | **%** |
| HIV testing services | 15 340 858 | 4.0% | HIV testing services | 1 052 147 | 0.3% |
| PMTCT | 731 858 | 0.2% | Prevention | 31 719 769 | 9.9% |
| Prevention--adolescents and youth | 12 660 068 | 3.3% | PMTCT | 731 858 | 0.2% |
| Prevention--general population | 15 387 215 | 4.0% | HIV/TB | 1 052 147 | 0.3% |
| Prevention--key populations | 3 672 486 | 1.0% | HIV treatment | 287 183 130 | 89.3% |
| HIV treatment | 287 183 130 | 74.6% | **TOTAL** | **321 739 051** | **100.0%** |
| TB | 6 984 078 | 1.8% |  |  | . |
| Multi-drug-resistant-TB | 4 131 643 | 1.1% |  |  |  |
| TB/HIV | 1 052 147 | 0.3% |  |  |  |
| RSSH | 27 801 970 | 7.2% |  |  |  |
| Programme management | 9 811 239 | 2.5% |  |  |  |
| **TOTAL** | **384 756 692** | **100.0%** |  |  |  |
|  |  |  |  |  |  |
|  |  |  |  |  |  |
| **Zimbabwe** |  |  | **HIV only** |  |  |
|  |  |  |  |  |  |
|  |  |  |  |  |  |
| **Module** | **US$** | **%** | **Module** | **US$** | **%** |
| HIV treatment | 308 448 396 | 71.4% | HIV testing services | 10 283 981 | 3.1% |
| Human resources for health | 17 376 000 | 4.0% | Prevention | 5 609 260 | 1.7% |
| HIV testing services | 23 074 707 | 5.3% | PMTCT | 1 795 260 | 0.5% |
| TB/HIV | 10 283 981 | 2,4% | HIV/TB | 10 283 981 | 3.1% |
| TB care and prevention | 10 498 438 | 2.4% | HIV treatment | 308 448 396 | 91.7% |
| Multi-drug-resistant-TB | 6 988 737 | 1.6% | **TOTAL** | **336 420 878** | **100.0%** |
| Procurement & supply management | 750 000 | 0.2% |  |  |  |
| PMTCT | 1 795 260 | 0.4% |  |  |  |
| Prevention for girls and women | 3 174 288 | 0.7% |  |  |  |
| Prevention for FSW and clients | 1 785 260 | 0.4% |  |  |  |
| Prevention for MSM | 649 712 | 0.2% |  |  |  |
| HMIS and M&E | 4 035 122 | 0.9% |  |  |  |
| Programme management | 43 029 474 | 10.0% |  |  |  |
| **TOTAL** | **431 889 375** | **100.0%** |  |  |  |
|  |  |  |  |  |  |

| **Tanzania** |  |  | **HIV only** |  |  |
| --- | --- | --- | --- | --- | --- |
|  |  |  |  |  |  |
|  |  |  |  |  |  |
| **Module** | **US$** | **%** | **Module** | **US$** | **%** |
| Prevention for general population | 21 544 036 | 4.8% | HIV testing services | - | 0.0% |
| Prevention for MSM | 2 990 439 | 0.7% | Prevention | 29 519 977 | 9.2% |
| Prevention for FSW and clients | 4 079 285 | 0.9% | PMTCT | 4 456 719 | 1.4% |
| Prevention for PWID | 5 832 073 | 1.3% | HIV/TB | 9 978 753 | 3.1% |
| Prevention for vulnerable groups (fisher-folk, miners) | 518 180 | 0.1% | HIV treatment | 277 950 025 | 86.3% |
| Prevention programs for adolescents and youth | 16 100 000 | 3.6% | **TOTAL** | **321 905 474** | **100.0%** |
| PMTCT | 4 456 719 | 1.0% |  |  |  |
| HIV treatment | 277 950 025 | 62% |  |  |  |
| TB/HIV | 9 978 753 | 2.2% |  |  |  |
| Programs to reduce human rights barriers | 853 830 | 0.2% |  |  |  |
| HMIS | 11 770 040 | 2.6% |  |  |  |
| PSM | 5 249 532 | 1.2% |  |  |  |
| Program Management | 22 537 576 | 5.0% |  |  |  |
| RSSH | 43 459 547 | 9.7% |  |  |  |
| TB care and prevention | 16 206 118 | 3.6% |  |  |  |
| Multi-drug-resistant-TB | 4 816 515 | 1.1% |  |  |  |
| **TOTAL** | **448 342 668** | **100.0%** |  |  |  |

**Legend:** FSW=female sex workers; HMIS=health management information systems; M&E=monitoring and evaluation; MSM=men-having-sex-with-men; PMTCT=prevention of mother-to-child transmission of HIV; PWID=people who inject drugs; RSSH=resilient and sustainable systems for health; VMMC=voluntary medical male circumcision.

**Table S3: Analysis of Global Fund PAAR Data for HIV**

| **Malawi** |  |  |
| --- | --- | --- |
|  |  |  |
| **Programme Area** | **Amount** | % |
| HIV treatment | 21 860 000 | 34,9% |
| HIV testing services | 9 700 000 | 15,5% |
| PMTCT | 2 720 000 | 4,3% |
| Prevention | 28 420 000 | 45,3% |
| **Total** | **62 700 000** | **100,0%** |
|  |  |  |
| **Tanzania** |  |  |
|  |  |  |
| **Programme Area** | **Amount** | % |
| HIV treatment | 6 576 844 | 30,4% |
| PMTCT | 3 921 708 | 18,1% |
| Prevention | 10 079 918 | 46,6% |
| TB/HIV | 706 600 | 3,3% |
| HMIS | 365 100 | 1,7% |
| **Total** | **21 650 170** | **100,0%** |
|  |  |  |
| **Zimbabwe** |  |  |
|  |  |  |
| **Programme Area** | **Amount** | % |
| HIV treatment | 66 987 154 | 46,4% |
| HIV testing services | 11 107 008 | 7,7% |
| PMTCT | 3 577 500 | 2,5% |
| VMMC | 5 698 778 | 3,9% |
| Prevention | 34 108 934 | 23,6% |
| TB/HIV | 4 897 537 | 3,4% |
| HMIS | - | 0,0% |
| Programme management | 17 906 030 | 12,4% |
| **Total** | **144 282 941** | **100,0%** |

**Legend:** FSW=female sex workers; HMIS=health management information systems; M&E=monitoring and evaluation; MSM=men-having-sex-with-men; PMTCT=prevention of mother-to-child transmission of HIV; PWID=people who inject drugs; RSSH=resilient and sustainable systems for health; VMMC=voluntary medical male circumcision.
